# Addition of SNAP to perinatal risk factors improves the prediction of bronchopulmonary dysplasia or death in critically ill preterm infants

**DOI:** 10.1186/1471-2431-13-138

**Published:** 2013-09-10

**Authors:** Yanhong Li, Jie Yan, Mengxia Li, Zhihui Xiao, Xueping Zhu, Jian Pan, Xiaozhong Li, Xing Feng

**Affiliations:** 1 Institute of Pediatric Research, Children’s Hospital affiliated to Soochow University, Suzhou, China; 2 Department of Nephrology, Children’s Hospital affiliated to Soochow University, Suzhou, China; 3 Department of Neonatology, Children’s Hospital affiliated to Soochow University, Suzhou 215003, China

**Keywords:** Adverse outcome, Bronchopulmonary dysplasia, Critically ill preterm infants, Mortality, Perinatal risk factors, Predictive test, SNAP (the score for neonatal acute physiology)

## Abstract

**Background:**

Bronchopulmonary dysplasia (BPD) is the most common serious pulmonary morbidity in premature infants. The score for neonatal acute physiology (SNAP) is a physiologic severity index for neonatal intensive care and correlates well with neonatal mortality and clinical outcomes. The prognostic value of the SNAP score for BPD in preterm infants remains to be clarified. The aim of the study was to determine whether SNAP can predict the development of BPD or death, and to investigate the contribution of SNAP to the predictive accuracy of other potential perinatal risk factors for the adverse outcome in critically ill preterm infants.

**Methods:**

We conducted a study in 160 critically ill preterm infants with less than 33 gestational weeks. The original SNAP score was prospectively calculated based on 28 items collected during the first 24 hours of admission. The potential perinatal risk factors were assessed during the first 72 hours of life. Major outcome measures were BPD and mortality before the time of BPD screening.

**Results:**

Of the 160 infants, 17 died and 41 developed BPD. The SNAP score was significantly associated with BPD or death (odds ratio [OR] =1.28; 95% confidence interval [CI], 1.16-1.41; *p* <0.001), even after adjustment for gestational age (OR =1.27; 95% CI, 1.13-1.41; *p* <0.001). High SNAP score was an independent predictor of BPD or death (area under the curve [AUC] =0.78; 95% CI, 0.70-0.85; *p* <0.001), with additional predictive value when combined with other perinatal risk factors. Multivariate regression analysis resulted in a final model, including SNAP, gestational age, apnea of prematurity, patent ductus arteriosus, and surfactant use as independent risk factors, with a higher predictive accuracy compared with individual components (AUC =0.92; 95% CI, 0.87-0.96; *p* <0.001).

**Conclusions:**

SNAP is associated with adverse outcome of BPD or death. High SNAP scores are predictive of BPD or death in critically ill preterm infants, and add prognostic value to other perinatal risk factors.

## Background

Bronchopulmonary dysplasia (BPD) is the most common serious pulmonary morbidity in premature infants [1-4]. Although there was a lack of universally acceptable accurate predictive models of BPD in clinical practice and research, it is widely acknowledged that BPD is a multi-factorial disorder, with low gestational age, low birth weight, lower Apgar scores, longer duration of oxygen exposure and assisted mechanical ventilation, and the presence of sepsis and patent ductus arteriosus (PDA) being important risk factors [5-10].

Illness severity scores have been widely used to adjust outcomes between populations for quality improvement and research purposes [11-15]. The score for neonatal acute physiology (SNAP) is a physiologic severity index for neonatal intensive care. SNAP is based on 28 items collected over the first 24 hours of life, and applicable to any infant admitted to a neonatal unit [11].

Previous studies have demonstrated that SNAP is a valid measure of illness severity at admission and important predictor of neonatal mortality and clinical outcomes [12,16-19]. However, to the best of our knowledge, no study has investigated the association between SNAP and the development of BPD. The predictive value of SNAP in critically ill preterm infants in relationship to BPD is currently unknown. In addition, mortality is a competing outcome for BPD. When identifying risk factors, the outcome of BPD/death should be used rather than BPD alone [9,20,21]. We hypothesized that in neonates SNAP could predict the development of BPD or death. The goal of this study was to investigate the contribution of SNAP to the predictive accuracy of other perinatal risk factors for the adverse outcome in critically ill preterm infants. It is clinically significant to evaluate additional predictive value by combining SNAP with other perinatal risk factors. Clinicians might be able to accurately predict BPD or death when taking account of the full and changing clinical picture of an infant.

## Methods

This study included preterm infants with less than 33 gestational weeks admitted to the neonatal intensive care unit (NICU) during the period from July 2010 to May 2012. Infants greater than 33 weeks of gestational age were not included because less than 1% of them developed BPD. The exclusion criteria were as follows: infants admitted to NICU after 24 hours of life, infants unexpectedly discharged or transferred to other departments or hospitals, and infants with severe congenital anomalies. The Institutional Ethics Review Board of the Children’s Hospital affiliated to Soochow University approved the study. Informed consent was obtained from the parents of the infants.

### Potential perinatal risk factors

To investigate the early prediction of BPD or death, the potential perinatal risk factors obtained during the first 72 hours of life were studied. Maternal data including chorioamnionitis, premature rupture of the membranes (PROM) >24 hours, gestational diabetes mellitus (GDM), pre-GDM, hypertension, pre-eclampsia, antenatal steroids, and mode of delivery, as well as neonatal data including gestational age, birth weight, gender, the Apgar scores, and delivery room resuscitation course were collected. Antenatal steroids were considered to have been administered if at least one dexamethasone injection was given 12 hours before delivery. Gestational age was calculated from the mother’s menstrual history and was confirmed by ultrasonography.

Clinical status, medication, and therapeutic interventions of the infants were recorded daily during the first 72 hours of life. We evaluated all infants for the presence of perinatal asphyxia, respiratory distress syndrome (RDS), transient tachypnea of the newborn, apnea of prematurity, pneumothorax, pulmonary hemorrhage, PDA, early onset sepsis, necrotizing enterocolitis (NEC), hypoxic-ischemic brain damage, intraventricular hemorrhage (IVH), and acute kidney injury.

In addition, we examined the use of postnatal steroids and surfactants at any time during the first 72 hours of life. Respiratory support, including oxygen, continuous positive airway pressure (CPAP), invasive mechanical ventilation, and the duration of therapies, were recorded. We assigned the respiratory support as none, nasal cannula, nasal CPAP, or invasive mechanical ventilation.

### The SNAP score

The original SNAP score was prospectively calculated for each infant based on 28 items collected during the first 24 hours of admission, including blood pressure, heart rate, respiratory rate, temperature, partial pressure arterial oxygen/fractional inspired oxygen (PaO_2_/FiO_2_) ratio, PaO_2_, partial pressure of carbon dioxide (PaCO_2_) in arterial blood, oxygenation index, hematocrit, white blood cell count, immature total ratio, absolute neutrophil count, platelet count, blood urea nitrogen, serum creatinine (Cr), urine output, serum indirect bilirubin, serum direct bilirubin, serum sodium, serum potassium, serum ionized calcium, serum total calcium, serum glucose, serum bicarbonate, arterial pH, seizure, apnea, and stool guaiac [11]. All the data were prospectively collected by trained graduate students under the supervision of the attending neonatologists and the chief of neonatology.

### Clinical outcomes

The outcome measures included the development of BPD or death before the time of BPD screening [20]*.*

### Definition of BPD

Infants were categorized as having mild, moderate, or severe BPD, according to the National Institutes of Health consensus definition of BPD [5,22]. Mild BPD was defined as receiving supplemental oxygen for ≥28 days but not at a postmenstrual age (PMA) of 36 weeks or at discharge; moderate BPD as receiving oxygen for ≥28 days plus treatment with <30% oxygen at 36 weeks’ PMA; and severe BPD as receiving oxygen for ≥28 days plus ≥30% oxygen and/or positive pressure at 36 weeks’ PMA.

### Diagnostic criteria and clinical indications

Apnea of prematurity was defined as cessation of breathing that lasts more than 15 seconds and is accompanied by bradycardia or hypoxia [23]. Diagnosis of PDA was based on echocardiogram and color Doppler test. PDA was considered as significant when the following criteria were met, as described previously: (a) a ductal diameter > 1.5 mm; (b) a left ventricular output index of more than 300 ml/kg/min; (c) left atrium to aortic root ratio greater than 1.5; (d) retrograde or absent diastolic flow in the cerebral anterior artery or in the descending thoracic aorta [24]. The criteria for the diagnosis of RDS were in accordance with the European consensus on the management of neonatal RDS in preterm infants. The diagnosis was confirmed on chest x-ray with a classical ‘ground glass’ appearance and air bronchograms. Infants with or at high risk of RDS were given a natural surfactant preparation [25,26]. Perinatal asphyxia was defined according to the following criteria: (1) pH ≤ 7.0 or base deficit ≥ 16 mmol/l in cord blood or during the first 1 hour after birth; or (2) If the pH was between 7.01 and 7.15, or the base deficit was between 10 and 15.9 mmol/L, or an arterial blood gas value was not available, either a 10 min-Apgar score ≤ 5 or assisted ventilation for ≥ 10 minutes from birth was required [27]. Once these criteria were met, all neonates underwent a standardized neurologic examination performed by neonatologists. Encephalopathy was defined as the presence of one or more signs in at least three of the following six categories (1) level of consciousness; (2) spontaneous activity; (3) posture; (4) tone; (5) primitive reflexes; and (6) autonomic nervous system signs [28]. Preterm infants who met the criteria for diagnosis of encephalopathy were considered as having hypoxic-ischemic brain damage, and diagnosis was confirmed by MRI examination. Diagnosis of IVH was based on the findings on head ultrasound and/or MRI according to the criteria of Papile et al. [29]. A head ultrasound was routinely performed in all preterm infants and in infants diagnosed with perinatal asphyxia. A brain MRI was conducted only in preterm infants who presented with signs and symptoms suggestive of encephalopathy or brain damage during the hospitalization, as described in our previous study [30]. The NEC diagnosis was made according to the modified Bell’s staging criteria. Infants with stage II or greater were defined as having NEC [31,32]. The diagnostic criteria of sepsis were in accordance with our previous study. Infants with positive clinical and/or laboratory screen and positive culture were defined as having sepsis [33]. EOS refers to sepsis presenting in the first 72 hours of life [34]. The diagnosis of AKI was based on serum Cr >1.5 mg/dl (132.6 μmol/l) and/or a ≥150% increase in serum Cr from baseline [33,35,36].

The indication for oxygen therapy in the newborn was PaO_2_ <50 mm Hg (6.6 kPa) or transcutaneous oxygen saturation <85% in room air, except in the presence of cyanotic congenital heart disease. Inability to maintain oxygenation with nasal cannula (FiO_2_ >60% to maintain PaO_2_ >50 mm Hg or arterial blood pH <7.25) or apnea unresponsive to stimulation is an indication for CPAP. The indications for mechanical ventilation were respiratory failure in newborns: PaO_2_ <50 mm Hg on FiO_2_ >60%, PaCO_2_ >60 mm Hg (8.0 kPa), arterial pH <7.20 or prolonged or repetitive unresponsive apnea associated with bradycardia or cyanosis.

### Statistical analysis

Statistical analyses were performed with SPSS 13.0. Bivariate and multivariate logistic regression was performed to identify potential perinatal risk factors associated with adverse outcome. Variables with *p* <0.2 in the bivariate analysis were entered into a backward stepwise multivariate regression model. Model fit was assessed with the Hosmer-Lemeshow goodness-of-fit test. A non-significant value for the Hosmer-Lemeshow Chi-square test suggests an absence of biased fit. Analysis of the area under the curve (AUC) of the Receiver Operating Characteristic (ROC) curve was constructed to assess the prognostic performance. The nonparametric method of Delong was used to compare significant difference between AUCs (Sigmaplot 10.0 software). Sensitivity and specificity were also determined. All probability values are two-sided. Differences with *p* values <0.05 were considered to be statistically significant.

## Results

A total of 160 infants were included in this study. During the time period of the study, 207 preterm infants with less than 33 gestational weeks were admitted to NICU during the first day of life. Forty-seven infants were excluded: 2 were diagnosed with severe congenital anomalies, 1 was transferred to another department, and 44 were unexpectedly discharged because of economic reasons.

Of the total 160 preterm infants, 17 died before the time of BPD screening. Among the 143 survived infants, 41 fulfilled the criteria for the diagnosis of BPD, including 25 with mild, 9 with moderate, and 7 with severe BPD. Patient characteristics of survivors without BPD and infants who died or developed BPD are shown in Table 1. In addition, there was no significant difference between included and excluded preterm infants with regards to gestational age (31.00 [25.86-32.86] *vs* 30.86 [24.00-32.86] weeks, *p* =0.797), birth weight (1460 [670–2320] *vs* 1550 [850–2300] g, *p* =0.285), gender (male/female: 104/56 *vs* 30/14, *p* =0.724), SNAP (10 [3-27] *vs* 9 [3-16], *p* =0.146), 1 min-Apgar score ( 8 [1-10] *vs* 8 [5-10], *p* =0.841), 5 min-Apgar score (9 [2-10] *vs* 8.5 [6-10], *p* =0.476), and oxygen therapy during the first 72 hours (*p* >0.05).

**Table 1:** Patient characteristics and bivariate analysis of perinatal risk factors for BPD or death*

	**Survival without BPD (n = 102)**	**BPD or Death (n = 58)**	**Odds ratio (95%)**^**#**^	***P *value**
Gestational age, weeks	31.86 [28.29-32.86]	29.29 [25.86-32.29]	0.41 (0.31-0.55)	<0.001
Birth weight, g	1570 [900–2320]	1330 [670–2100]	0.81 (0.72-0.90)^a^	<0.001^b^
Gender, male/female	67/35	37/21	1.09 (0.55-2.13)	0.809
Small for gestational age (<10%), n (%)	12 (11.8)	9 (15.5)	1.38 (0.54-3.50)	0.500
Apgar score, 1 min	8 [1-10]	7 [1-10]	0.90 (0.77-1.04)	0.139
Apgar score, 5 min	9 [3-10]	8.5 [2-10]	0.76 (0.63-0.93)	0.007^c^
SNAP	8 [3-26]	13 [4-27]	1.28 (1.16-1.41)^d^	<0.001^c^
Apnea of prematurity, n (%)	12 (11.8)	18 (31.0)	3.23 (1.42-7.33)	0.005^b^
Patent ductus arteriosus, n (%)	1 (1.0)	8 (13.8)	16.33 (1.99-134.2)	<0.001^c^
Respiratory distress syndrome, n (%)	45 (44.1)	50 (86.2)	8.89 (3.68-21.49)	<0.001^c^
Hypoxic-ischemic brain damage, n (%)	7 (6.9)	2 (3.4)	0.49 (0.10-2.43)	0.382
Intraventricular hemorrhage, n (%)	0 (0)	3 (5.2)	3E + 009	0.999
Early-onset sepsis, n (%)	4 (3.9)	2 (3.4)	0.88 (0.16-4.97)	0.887
Respiratory support^e^			2.95 (1.89-4.61)	<0.001^c^
Oxygen therapy, n (%)	85 (83.3)	57 (98.3)	9.39 (1.20-73.39)	0.033^b^
CPAP, n (%)	45 (44.1)	30 (51.7)	1.16 (0.60-2.25)	0.659
Mechanical ventilation, n (%)	21 (20.6)	33 (56.9)	4.97 (2.45-10.08)	<0.001^c^
Surfactant, n (%)	35 (34.3)	46 (79.3)	7.12 (3.34-15.17)	<0.001^c^
Postnatal steroid, n (%)	23 (22.5)	20 (34.5)	1.81 (0.89-3.69)	0.104
Antenatal steroid, n (%)	11 (10.8)	5 (8.6)	0.78 (0.26-2.36)	0.658
Pre-eclampsia, n (%)	14 (13.7)	7 (12.1)	0.86 (0.33-2.28)	0.766
Premature rupture of the membranes (>24 h), n (%)	14 (13.7)	10 (17.2)	1.31 (0.54-3.17)	0.550

Values are median [min-max range]; *BPD* bronchopulmonary dysplasia, *CI* confidence interval, *CPAP* continuous positive airway pressure, SNAP the score for neonatal acute physiology.

*Potential perinatal risk factors obtained during the first 72 hours of life were studied. ^#^Data from all preterm infants (n =160) were analyzed to predict BPD or death (n =58). Preterm infants who died or developed mild, moderate, or severe BPD were combined into one group.

^a^Odds ratio represents the increase in risk per 100 g increase in birth weight. ^b^The association did not remain significant after controlling for gestational age. ^c^The association remained significant after controlling for gestational age. ^d^Odds ratio per 1-point increase in SNAP. ^e^Respiratory support was assigned as none, nasal cannula, nasal CPAP, or invasive mechanical ventilation.

### Bivariate analysis of risk factors for the development of BPD or death

Data from all preterm infants were analyzed to predict BPD or death. Preterm infants with mild BPD were included into the group of BPD or death. There was no association between the severity of BPD and BPD or death (*p* =0.996).

Preterm infants with adverse outcome of BPD or death were more likely than controls to have a lower gestational age at delivery, lower birth weight, lower 5 min-Apgar score, and higher SNAP score (Table 1). The impact of SNAP (Odds ratio [OR] per 1-point increase = 1.27, 95% confidence interval [CI] 1.13-1.41, *p* <0.001) and 5 min- Apgar score (OR 0.72, 95% CI 0.57-0.91, *p* =0.005) persisted when controlling for gestational age. Birth weight, however, did not remain associated with BPD or death when adjusted for gestational age. Gender and 1 min- Apgar score were not associated with BPD or death at the unadjusted bivariate level. Furthermore, SNAP (OR 1.21, 95% CI 1.07-1.39, *p* =0.004), 5 min-Apgar score (OR 0.59, 95% CI 0.43-0.81, *p* =0.001) and gestational age (OR 0.37, 95% CI 0.23-0.58, *p* <0.001) remained associated with BPD or death after controlling for the severity of BPD.

Among infants with BPD or death, apnea of prematurity, RDS, and PDA were observed more frequently than in the control group. The association of RDS and PDA with BPD or death remained significant after controlling for gestational age. In contrast, the presence of early onset sepsis, hypoxic-ischemic brain damage, IVH, NEC or pneumothorax was not associated with BPD or death.

Preterm infants with BPD or death were much more likely than controls to have received oxygen therapy and to be exposed to mechanical ventilation*.* Although we did not find an association between nasal CPAP and BPD or death, there was a significant association of respiratory support included nasal cannula, nasal CPAP, and invasive mechanical ventilation with BPD or death.

Moreover, BPD or death cases had more surfactant treatment than controls. After adjustment for gestational age, surfactant use was associated with a seven-fold increased risk of BPD or death (OR 6.71, 95% CI 2.70-16.63, *p* <0.001). There was no association between pre-eclampsia, PROM, caesarean section or antenatal steroid use and BPD or death.

### Multivariate analysis of risk factors for the development of BPD or death

Variables with *p* <0.2 in the bivariate analysis were entered into a backward stepwise multivariate logistic regression analysis to identify the factors independently associated with increased risk of BPD or death in critically ill preterm infants. The final model retained gestational age, SNAP, the presence of apnea of prematurity and PDA, and surfactant use as significant predictors, with 5 min- Apgar score as a confounder (*p* =0.071) (Table 2). The Hosmer-Lemeshow goodness-of-fit test for the final model was not significant (*p* =0.333), indicating that the model fits the data adequately.

**Table 2:** **Multivariate logistic regression final model predicting BPD or death**^**a**^

	**Odds ratio**^**b**^	**95% CI**	***P *value**
Gestational age	0.38	0.25-0.57	<0.001
SNAP	1.19^c^	1.04-1.32	0.010
Apnea of prematurity	4.89	1.18-20.36	0.029
Patent ductus arteriosus	16.51	1.08-252.49	0.044
Surfactant use	7.99	2.33-27.47	0.001
Apgar score, 5 min	0.77	0.59-1.02	0.071

*BPD* bronchopulmonary dysplasia, *CI* confidence interval, *SNAP* the score for neonatal acute physiology.

^a^Variables with *P* <0.2 in the unadjusted analysis (Table 1) were entered into a backward stepwise multivariate logistic regression analysis. ^b^Data from all preterm infants (n =160) were analyzed to predict BPD or death (n =58). ^c^Odds ratio per 1-point increase in SNAP.

The *p* value of the Hosmer-Lemeshow goodness-of-fit test for the final model was 0.333.

### SNAP as predictor for BPD or death

Based on the above-mentioned association between SNAP and adverse outcome of BPD or death, SNAP could be a useful prognostic predictor in critically ill preterm infants. To assess the potential to predict BPD or death, we compared the predictive value of SNAP with other prognostic predictors demonstrated by multivariate regression analysis (Table 3). Gestational age and SNAP appeared to be significant predictors, and achieved AUC of 0.83 (95% CI 0.76-0.89, *p* <0.001) and 0.78 (95% CI 0.70-0.85, *p* <0.001) respectively, for predicting the development BPD or death. When combining SNAP and gestational age, the predictive performance improved (AUC 0.88, 95% CI 0.84 to 0.94, *p* <0.001) over that of gestational age alone, but not reaching statistical significance (*p* =0.178).

**Table 3:** Predicting performance of SNAP and other risk factors for development of BPD or death

	**AUC**	**95% CI**	***P *value**
Low gestational age	0.83	0.76-0.89	<0.001
SNAP	0.78	0.70-0.85	<0.001
Surfactant use	0.72	0.64-0.80	<0.001
Apnea of prematurity	0.59	0.50-0.69	0.050
Patent ductus arteriosus	0.57	0.47-0.66	0.174
SNAP, combined with gestational age	0.88	0.84-0.94	<0.001
SNAP, combined with surfactant use	0.81	0.75-0.88	<0.001
SNAP, combined with gestational age and surfactant use	0.90	0.85-0.95	<0.001
Combination of 5 risk factors^a^	0.92	0.87-0.96	<0.001
*P* value (comparison of the difference between AUCs)			
*p* = 0.352 (between SNAP and gestational age)			
*p* = 0.178 (between SNAP combined with gestational age and gestational age alone)			
*p* = 0.023 (between SNAP combined with gestational age and SNAP alone)			
*p* = 0.026 (between combination of 5 risk factors and gestational age alone)			
*P* = 0.002 (between combination of 5 risk factors and SNAP alone)			
*p* = 0.362 (between combination of 5 risk factors and SNAP combined with gestational age)			

*AUC* area under the receiver-operating-characteristic curve, *BPD* bronchopulmonary dysplasia, *CI* confidence interval, *SNAP* the score for neonatal acute physiology.

^a^Five risk factors: gestational age, SNAP, surfactant use, apnea of prematurity, and patent ductus arteriosus.

**Figure 1:**
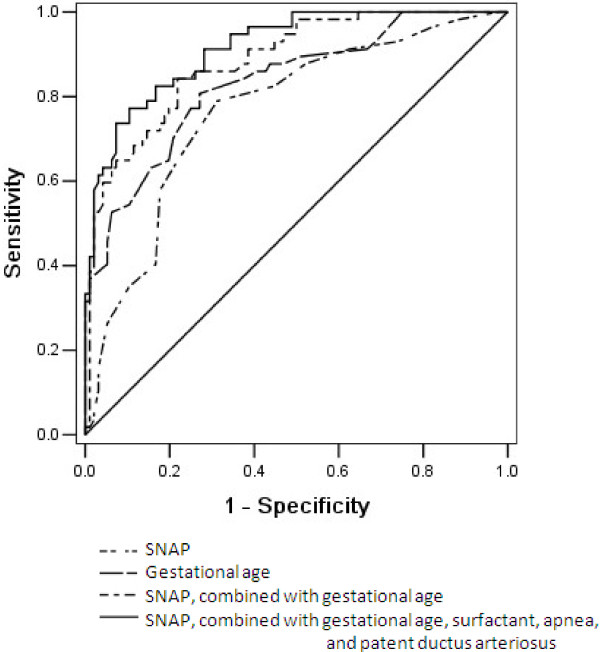
**Receiver operating characteristic curves for the ability of the score for neonatal acute physiology (SNAP), gestational age and the models to predict the development of bronchopulmonary dysplasia or death in critically ill preterm infants with less than 33 gestational weeks (n =160).** The area under the receiver operating characteristic curve for gestational age, SNAP, SNAP combined with gestational age, and SNAP combined with gestational age, apnea of prematurity, patent ductus arteriosus, and surfactant use were 0.83, 0.78, 0.88 and 0.92, respectively, with a Hosmer-Lemeshow goodness-of-fit *p* value >0.05.

Multivariate logistic regression analysis resulted in a superior final model containing variables of SNAP, gestational age, the presence of apnea of prematurity and PDA, and surfactant use as independent risk factors. With an AUC of 0.92 (95% CI 0.87-0.96, *p* <0.001), the prognostic accuracy of the final model was superior to the accuracy of the other predictors. By using the nonparametric method of Delong, the prognostic performance of the final model was significantly better than gestational age (*p* =0.026) or SNAP (*p* =0.002) alone. Figure 1 shows the ROC curves and AUC for the final model and for SNAP and gestational age.

The sensitivity and specificity of risk factors for the development of BPD or death are shown in Table 4. SNAP displayed sensitivity of 79% and specificity of 71% to predict BPD or death at the optimal cut-off score of 10.5, and the positive and negative likelihood ratios were 2.7 and 0.29, respectively. Gestational age displayed sensitivity 81% and specificity 73% at the optimal cut-off value of 31 weeks for predicting the development of BPD or death, with the positive and negative likelihood ratios of 3.0 and 0.26, respectively. When combining the variables of SNAP and gestational age, the sensitivity and specificity increased to 84% and 77%, with the positive and negative likelihood ratios of 3.8 and 0.20, respectively. Meanwhile, when we combined the variables of SNAP, gestational age, apnea of prematurity, PDA, and surfactant use in the final model, specificity increased to 90%, and positive likelihood ratio increased to 7.4.

**Table 4:** Predictive characteristics of SNAP and other risk factors for BPD or death

	**Cut-off value**	**Sensitivity**	**Specificity**	**PPV**	**NPV**	**LR+**	**LR-**
Gestational age	31 weeks	81%	73%	75%	79%	3.0	0.26
SNAP score	10.5	79%	71%	73%	77%	2.7	0.29
SNAP, combined with gestational age		84%	77%	79%	83%	3.8	0.20
Combination of 5 risk factors^a^		77%	90%	88%	80%	7.4	0.25

*BPD* bronchopulmonary dysplasia, *LR +* likelihood ratio positive, *LR-* likelihood ratio negative, *NPV* negative predictive value, *PPV* positive predictive value, *SNAP* the score for neonatal acute physiology.

^a^Five risk factors: gestational age, SNAP, surfactant use, apnea of prematurity, and patent ductus arteriosus.

## Discussion

Our data demonstrate that the SNAP score based on 28 items collected during the first 24 hours of admission to NICU is significantly associated with the development of BPD or death in critically ill preterm infants with less than 33 gestational weeks. High SNAP score is predictive of BPD or death, and adds predictive value to other potential perinatal risk factors in this population.

The present study first confirmed the known strong association between low gestational age and BPD [6,7,9]. Infants with lower gestational age have worse outcomes. The gestational age displayed the best predictive performance, when compared with other perinatal risk factors determined by bivariate analysis. Our study is the first to compare the predictive value of candidate perinatal risk factors obtained during the first 72 hours of life, for the early prediction of BPD or death. Our findings are in agreement with a large multicenter study, which has reported that the gestational age was the most predictive information for BPD risk on postnatal days 1 and 3 in infants of 23–30 weeks’ gestational age [9].

The major finding in the study was that the SNAP score was significantly associated with adverse outcome of BPD or death. For each point increment in the SNAP score, the odds ratio for predicting BPD or death was 1.28. The SNAP score, based on 28 items from a variety of sources including every body system and selected blood test results, reflects the full clinical picture of an infant during the early postnatal period. Previous studies suggest that considering the full clinical picture of an infant is likely beneficial in the prediction of neonatal mortality and morbidity [12,16-19]. One study evaluated the usefulness of SNAP version II (SNAP-II) to predict BPD [37], suggesting SNAP-II predicts chronic lung disease in the NICU. SNAP-II is a simpler version of SNAP and reduces the number of variables to six [12]. This previous study, however, excluded infants who died, which is a competing outcome for BPD.

The SNAP score and gestational age contributed independently to the risk of BPD or death. Although the predictive accuracy of SNAP for BPD or death (AUC of 0.78) was not better than that of gestational age (AUC of 0.83), the addition of SNAP improved BPD or death prediction of gestational age (AUC of 0.88), increasing the contribution of gestational age to BPD or death. This suggests that SNAP was not a confounder here, but was probably an effect modifier. In addition, the combination of gestational age, apnea of prematurity, PDA, surfactant use and SNAP appeared to be the best predictive model for BPD or death in critically ill preterm infants, with an AUC of 0.92. Our data indicate that SNAP, as a measure of newborn illness severity, could add predictive value to other previously described perinatal risk factors for BPD or death.

Previously identified risk factors for BPD, including gestational age, apnea of prematurity, PDA, and surfactant use, were included in our final model [5,7,9,38]. Surfactant therapy is a standard of care for infants at risk of RDS. Although current evidence is insufficient to support surfactant treatment strategies for the prevention of BPD [4,39], the surfactant with brief ventilation as part of the intubation surfactant extubation approach was found to reduce the rates of BPD [40]. Surfactant use included in the model is likely not causal for BPD or death but reflective of respiratory illness or management. Previously described risk factors, including birth weight, male gender, sepsis, oxygen therapy, and mechanical ventilation, were not included in the model [5,7,9,21,41]. None of these factors improved prediction of the risk of BPD or death after adjustment for the five critical risk factors. The comparison of the prognostic value of SNAP to the predictive ability of known risk factors on the adverse outcome of critically ill preterm infants is a novel aspect of this work. Our findings suggest that the additional predictive value of combining SNAP with known perinatal risk factors improves the quantification of the risk for prediction of neonatal mortality and morbidity.

Neonatal care has changed dramatically over the past decades, and the improvement in antenatal corticosteroid treatment, surfactant replacement therapy and ventilator support greatly reduced the severity of long-term respiratory morbidity and mortality. Despite that, the risk of developing BPD remains high [42]. The overall aim of the clinical research is to identify novel preventive and therapeutic strategies for BPD. It has been documented that vitamin A supplementation in extremely low birth weight infants and caffeine therapy for apnea of prematurity are effective in reducing the incidence of BPD [39]. Our findings will contribute to identifying preterm infants at high risk of BPD who could benefit the most from the prophylactic administration of vitamin A and caffeine during the early period of life. In addition, our study focused on the early postnatal period, which is clinically relevant. Previous studies suggested that the benefits of postnatal treatment strategies might be dependent on the baseline risk of BPD or death [9,43].

There were several limitations to our study. First, this was a single-center study. Our results might be biased due to the small sample size. There was a wide confidence interval for odds ratio of apnea of prematurity, PDA and surfactant use for BPD or death in the final model, which might be in part attributed to a low incidence related to the small sample size. Second, we excluded 44 infants who were unexpectedly discharged before the time of BPD screening because of economic reasons, although demographic and clinical characteristics of infants included in the analyses were comparable with those being excluded, as shown in the results. Furthermore, the ratio of male to female in the study population was 1.9:1 (104/56). During the study period, the gender ratio of male to female in neonates admitted to our unit of neonatology was 1.5:1. We were not able to determine the effects of gender differences. These factors may lead to the potential of selection bias and limit the generalizability of our results to similar patient populations in other NICUs. The results of the study should be confirmed in a multicenter study. Third, SNAP, unlike SNAP-II and the clinical risk for babies score (CRIB), is cumbersome to use in clinical practice. SNAP-II is scored within the first 12 hours of admission, and thus limited in practical use. The CRIB score is only created to predict mortality for infants born at less than 32 weeks gestation at birth [12].

## Conclusions

In the study, high SNAP scores were significantly associated with increased risk for BPD or death in critically ill preterm infants with less than 33 gestational weeks. SNAP is predictive of BPD or death, with additional prognostic value when used in conjunction with other perinatal risk factors. The combination of SNAP score with gestational age, apnea of prematurity, PDA, and surfactant use appears to be the best predictive model for BPD or death during the early postnatal period in this population. Large studies are needed to further explore the role of the SNAP score for prediction of BPD or death in neonates.

## Abbreviations

AUC: Area under the receiver-operating-characteristic curve; BPD: Bronchopulmonary dysplasia; CI: Confidence interval; CPAP: Continuous positive airway pressure; CRIB: Clinical risk for babies score; GDM: Gestational diabetes mellitus; IVH: Intraventricular hemorrhage; NEC: Necrotizing enterocolitis; NICU: Neonatal intensive care unit; OR: Odds ratio; PDA: Patent ductus arteriosus; PMA: Postmenstrual age; PROM: Premature rupture of the membranes; RDS: Respiratory distress syndrome; ROC: Receiver operating characteristic; SNAP: The score for neonatal acute physiology; SNAP-II: SNAP version II.

## Competing interests

The authors declare that they have no competing interests.

## Author’s information

Xiaozhong Li and Xing Feng share corresponding authorship.

## Authors’ contributions

YH Li participated in study design, protocol development and performance, data analysis, interpretation of data and writing of the manuscript. J Yan, MX Li and J Pan carried out the clinical data collection and data analysis. ZH Xiao and XP Zhu participated in clinical evaluation for diagnosis and interpretation of data. XZ Li and X Feng participated in the design of the study and coordination and helped to draft the manuscript. All authors read and approved the final manuscript.

## Pre-publication history

The pre-publication history for this paper can be accessed here:

http://www.biomedcentral.com/1471-2431/13/138/prepub
